# 3for1 intervention program—short-term psychotherapy, job coaching, and peer support for unemployed individuals facing psychological distress: a community case study

**DOI:** 10.3389/fpubh.2026.1805157

**Published:** 2026-05-26

**Authors:** Sophia Helen Adam, Sonja Tomašković, Julia-Maria Koch, Hannah Weiwadel, Melanie Gantner, Nick Werner, Oliver Schmitt, Marco Cemernjak, Doris Schuderer, Jörn von Wietersheim, Miriam Mehler, Rebecca Erschens

**Affiliations:** 1 Department for Psychosomatic Medicine and Psychotherapy, University Hospital Tübingen, Tübingen, Germany; 2 Department for Psychosomatic Medicine and Psychotherapy, University Hospital Ulm, Ulm, Germany

**Keywords:** job coaching, mental health, peer support, psychological distress, short-term psychotherapy, unemployment

## Abstract

This community case study examines the 3for1 intervention program, a multidisciplinary intervention designed to support individuals affected by unemployment experiencing psychological distress. The program integrates short-term psychotherapy, job coaching based on the approach of Individual Placement and Support (IPS), and peer support. Thus, individuals affected by complex and interrelated psychosocial challenges can find support through individualized, low-threshold care. Two participants are presented: Participant A regained employment, Participant B primarily improved mental health outcomes. In both cases, psychotherapy mobilized personal resources and supported decision-making, job coaching facilitated re-employment and peer support enhanced self-efficacy. Close multidisciplinary collaboration enabled tailored support. Participants’ trajectories indicate the value of individualized, cross-sector approaches in addressing barriers to labor market participation for individuals affected by psychological distress. Structural challenges underscore the need for longer-term, integrated support for individuals with complex psychosocial needs.

## 1 Introduction

This community case study illustrates the application of the 3for1 intervention program offered by six job centers in south Germany funded by the German Federal Ministry of Labor and Social Affairs (1). Job centers in Germany are government agencies responsible for administering basic income support and providing employment services as part of the country’s social welfare system. The 3for1 intervention program addresses the challenges faced by individuals affected by unemployment and psychological distress, as they are less likely to find reintegration into the primary labor market (2). This lack of reintegration hinders their ability to secure competitive employment and burdens the German social insurance system (3). The 3for1 intervention program consists of short-term psychotherapy, Individual Placement and Support (IPS)-based job coaching (4, 5), and peer support. It aims so reintegrate job center clients in the primary labor market and improve their mental health.

Existing programs targeting job center clients have either focused on job coaching and training (“first train, then place”) (6), or primarily addressed mental health issues (7–10). The first train, then place approach aims to prepare individuals for employment through pre-vocational training and stabilization before entering the labor market. While this approach may provide a structured environment and support skill development, it can be criticized for delaying labor market integration and for limited transferability of acquired skills to real work settings.

In contrast, approaches such as IPS follow a first place, then train principle, emphasizing rapid placement in competitive employment combined with on-the-job support. This model has shown strong evidence for improving employment outcomes, particularly among individuals with severe mental health conditions, but may require substantial coordination and ongoing support, and may not be equally suitable for all individuals depending on their diagnosis and the specific service context in which it is implemented (11). Research indicates that programs that address both, mental health concerns and reintegration into the primary labor market, yield more effective results (9, 12), for an overview see (7).

Hence, the 3for1 intervention program aims to contribute to the ongoing discourse on interdisciplinary approaches for labor market reintegration of individuals with complex psychosocial needs. The aim of this community case study is twofold: to illustrate the structure of the 3for1 intervention program and to demonstrate the application of the three intervention components (short-term psychotherapy, IPS-based job coaching, and peer support), provided by case studies of two clients. Particular attention is given to the collaborative processes between intervention providers, the challenges encountered, and the individual adaptations required to support clients effectively.

## 2 Context (setting and population)

There is a strong correlation between unemployment and disadvantaged mental health outcomes, with the two conditions mutually reinforcing each other in a vicious circle (13–15). Loss of employment can lead to feelings of hopelessness and impaired mental health (16, 17), while also contributing to various physical health struggles, such as poor cardiovascular health and cancer, and increased risks of mortality, including suicide (18–24). At the same time, individuals experiencing psychological distress may find it particularly difficult to maintain employment, which can further exacerbate life management problems such as keeping time structure, engaging in social contact and remaining an active life (3, 14, 25). It is therefore of crucial importance to recognize these interconnected challenges and to offer adequate support.

Individuals targeted by the 3for1 intervention program typically experience a broad range of psychosocial challenges that extend beyond a single diagnostic category. These challenges include clinically relevant mental health issues, such as depression and anxiety, but also subthreshold mental health problems that significantly impair daily functioning without necessarily meeting formal diagnostic criteria. In addition, many individuals face reduced self-efficacy regarding job search and employment, pronounced fears related to returning to work, and experiences of both public stigma and self-stigma associated with unemployment and mental health problems. Also, barriers to help-seeking are common and may include limited mental health literacy, uncertainty about available services, and structural difficulties in navigating the health care and social security systems. These psychological challenges are closely intertwined with social and functional difficulties. Affected individuals often struggle with structuring their daily lives, maintaining social participation, and managing administrative demands. At the same time, they may encounter fragmented support systems and bureaucratic barriers, which can delay or prevent access to adequate care.

Taken together, these factors result in complex and interrelated psychosocial challenges that simultaneously affect mental health, social integration, and the ability to retain employment. Thus, the 3for1 intervention program is designed to address these challenges through a multi-level, individualized approach that integrates mental health support, vocational rehabilitation, and peer-based guidance (1).

Against this background, it is essential to consider the structural support context. In Germany, people who are unable to support themselves financially are eligible to apply for basic income support, which is administered by local job centers. Alongside financial assistance, job center *clients* are offered a potentially wide range of support services to facilitate their reintegration into the labor market. However, support for individuals with mental health challenges in the German social security system is often fragmented. While job centers are mainly responsible for promoting employability and labor market reintegration, the health care system focuses on treatment and recovery, and other institutions, such as the pension insurance system, may become involved if individuals are considered unable to work. At the same time, untreated or insufficiently addressed mental health conditions are highly prevalent among job center clients and represent a major barrier to labor market participation (26). Many individuals have complex and overlapping needs that require coordination across these systems. In practice, however, collaboration between services can be limited due to differing institutional responsibilities and regulatory frameworks. This can result in delays in accessing appropriate support and create substantial barriers for both clients and professionals navigating the system (27, 28). Within this complex system, job centers play a central role in supporting individuals’ labor market reintegration. However, job center employees often find it difficult to recognize mental health problems, for which they are typically not trained (29).

This is where the 3for1 intervention program takes action. It aims to support individuals experiencing subclinical and clinically relevant psychological distress within job centers, with the goal of promoting mental health recovery in daily life and facilitating access to and retention of employment in the primary labor market. The project achieves these goals by providing individualized support tailored to participants’ specific needs.

## 3 Programmatic elements

The study was designed as a multi-center, non-randomized controlled trial with time-based allocation to either a treatment-as usual (TAU) control group (CG) or the intervention group (IG) and registered in the German Clinical Trials Register (DRKS00029002). Potential participants of the 3for1 intervention program were recruited via employment advisors in the six collaborating job centers in the southwest of Germany and screened for eligibility, including the assessment of psychological distress using the Kessler Psychological Distress Scale (K6; range: 0–24 (30)) where participants had to display at least moderate psychological distress indicated by a score of nine or greater.

Both groups received TAU within the job center setting, while participants in the IG were additionally offered access to the 3for1 intervention program. Eligible participants were informed about the study procedures by research staff, provided written informed consent, and completed a baseline assessment prior to entering the intervention phase. Participants in the IG received access to 3for1 over a total period of 12 months. The intervention followed a flexible and participant-centered approach: participants could choose between the three components (short-term psychotherapy, IPS-based job coaching, and peer support) and decide independently if, when and how to engage with them. Components could be initiated concurrently or consecutively, paused, or discontinued at any time, depending on individual needs and preferences and not all of the three-component needed to be used. Further details regarding recruitment, time frame, inclusion- and exclusion criteria can be found in the study protocol (1).

Short-term psychotherapy follows an integrative psychotherapeutic approach based on the well-established model of psychotherapeutic consultation in the workplace (31). Participants are offered up to 10 sessions, along with three optional additional booster sessions. A key feature is that individuals were able to seek counseling without long waiting times (32, 33) and without a formal record of utilization with their health insurance. Sessions are delivered by clinically trained psychotherapists or psychologists affiliated with the project and typically last up to 50 min, corresponding to standard outpatient psychotherapy sessions in the German health care system. Sessions could take place either in dedicated rooms within the job center or at the respective university outpatient clinics, allowing participants to choose a setting that ensured sufficient distance from the job center environment if desired. Appointments were arranged flexibly and individually, depending on participants’ needs and available capacities. The intervention is designed as a low-threshold service, allowing participants to access support without long waiting times and without formal registration within the health insurance system. Initial sessions focus on comprehensive assessment and goal clarification, as well as promoting motivation for change. Subsequent sessions are tailored to individual needs and may include psychoeducation, development of a biopsychosocial understanding of mental health problems, resource-oriented work, support in structuring daily routines, and the promotion of self-efficacy, problem-solving skills, and social and emotional competencies. In addition, the indication for further treatment is assessed throughout the intervention, and participants may be supported in accessing outpatient or inpatient care if needed. Booster sessions may be offered to support relapse prevention, maintain treatment gains, or bridge waiting times until further treatment begins.

IPS-based job coaching (4, 5) implements the principle of “first place, then train”. Individuals are placed in the primary labor market as soon as possible and then trained in the workplace. Core IPS principles include rapid job search, a focus on competitive employment, and individualized, client-centered support integrated with mental health care. In the present intervention, IPS principles were followed and partially adapted to the specific context of job centers and to a broader target group including individuals with varying levels of psychological distress. Therefore, the intervention represents a context-sensitive application of the IPS approach within a multidisciplinary support framework. A total of 25 appointments were available with the job coach. If participants managed to find employment, the job coaching could continue until the end of the 12-month period to support participants during the transition back into the working environment.

Peer support was delivered by peer navigators, for the use of this project defined as individuals with lived experience of mental illness and life crises (1, 34). Many peer navigators within the 3for1 intervention program also had experienced periods of unemployment, reduced earning capacity, or receipt of disability benefits. In contrast to professional health or social care providers, peer navigators are considered “experts by experience,” whose role is grounded in shared lived experience rather than formal clinical training. Thus, peer navigators were recruited based on their lived experience with mental health challenges and/or unemployment. A formal qualification as a peer-support worker (e.g., EX-IN or comparable training) was not a prerequisite for this role. However, where such qualifications were present, they were integrated into the respective roles and competency profiles of the peer navigators. They received ongoing supervision within the project and were financially compensated on a part-time, marginal employment basis. In addition, regular supervision and team meetings were provided to support peer navigators in managing the emotional demands of their role. Each local intervention team included four peer navigators, each employed on a marginal part-time basis (approximately 7 h per week). All peer navigators worked according to a manualized approach [adapted from (35), received a standardized 1.5-day in-person training].

A central component of peer support is the disclosure of lived experience with mental health challenges in order to foster mutual understanding, reduce perceived stigma, and build a trusting relationship with participants (34, 36). Peer navigators support participants both emotionally and instrumentally in navigating everyday challenges, particularly within the health care system, social security system, and interactions with official authorities. This may include accompanying participants to appointments (e.g., with health professionals, job centers, or debt counseling services), supporting activation in daily life (e.g., engaging in physical activity or initiating new routines), and facilitating the development of social networks (e.g., through self-help groups).

An important element of the peer-support component was the collaborative definition of individual goals, which could vary depending on participants’ needs. These ranged from emotional support and activation of personal resources in daily life to more practical forms of support, such as guidance in dealing with administrative tasks (e.g., completing forms). Such activities were understood as part of supporting participants in navigating perceived everyday challenges and did not replace professional services such as social work or employment assistance.

Peer support follows the principle of working on equal footing, with a focus on empowerment and supporting individuals in regaining control over their lives and recovery processes (37). Within the intervention, peer support was available without predefined limits throughout the 12-month period and was provided in a flexible, needs-based manner.

## 4 Utilization of all three interventional components and selection of participants

The selection of the case studies followed several criteria. Participants were recruited toward the end of the intervention period, in line with the overall project and publication timeline. In collaboration with the treating staff, individuals were selected who had engaged with all three intervention components, as not all participants made use of each module. Furthermore, we aimed to include cases from both study sites and to select participants whose trajectories allowed for the illustration of meaningful development, interactional dynamics, and interconnections between the intervention components. Additional practical considerations included participants’ availability for follow-up contact and their willingness to provide informed consent for the use of their data in a scientific case study, which further limited the pool of eligible cases. Importantly, prior experience with psychiatric care and the job center system was common within the study population and therefore reflects typical rather than exceptional characteristics of the target group. Accordingly, the selected cases should be understood as illustrative examples of broader patterns observed within the intervention rather than as extreme or atypical cases.

### 4.1 Participant A

#### 4.1.1 Participant demographics for Participant A

Participant A is a woman in her late 40s who joined the 3for1 intervention program in mid-2023. Born in Eastern Europe, she moved to Germany as a child and holds dual nationality. She has three siblings and is married. They have three children (aged between 16 and 20) who all still live at home. She holds a commercial qualification but has mostly worked in childcare as a part time employee. She was dismissed from her last job as a commercial clerk 4 years ago. No reasons were given for her dismissal. She described her partner as emotionally unsupportive. Though in divorce proceedings, she still lived with him at the start of the intervention and faced financial hardship. She has received treatment at a psychiatric day clinic 6 months before the study.

#### 4.1.2 Diagnostic findings

The initial assessment revealed notable psychopathological symptoms. Concentration and memory were found impaired. She focused on negative experiences with her estranged husband and also voiced concerns about her future and finances. A moderately depressed mood and sleep disturbances were reported. No suicidal tendencies were reported. Prior to joining the 3for1 intervention program, she had been on sick leave for 6 months. She reported headaches and back pain from herniated discs. Health status was assessed via questionnaires during inclusion. Further information can be found in the study protocol (1). Interviews and questionnaires revealed symptoms of anxiety, depression, reduced quality of life, and high stress.

#### 4.1.3 Interventions

**Figure 1:**
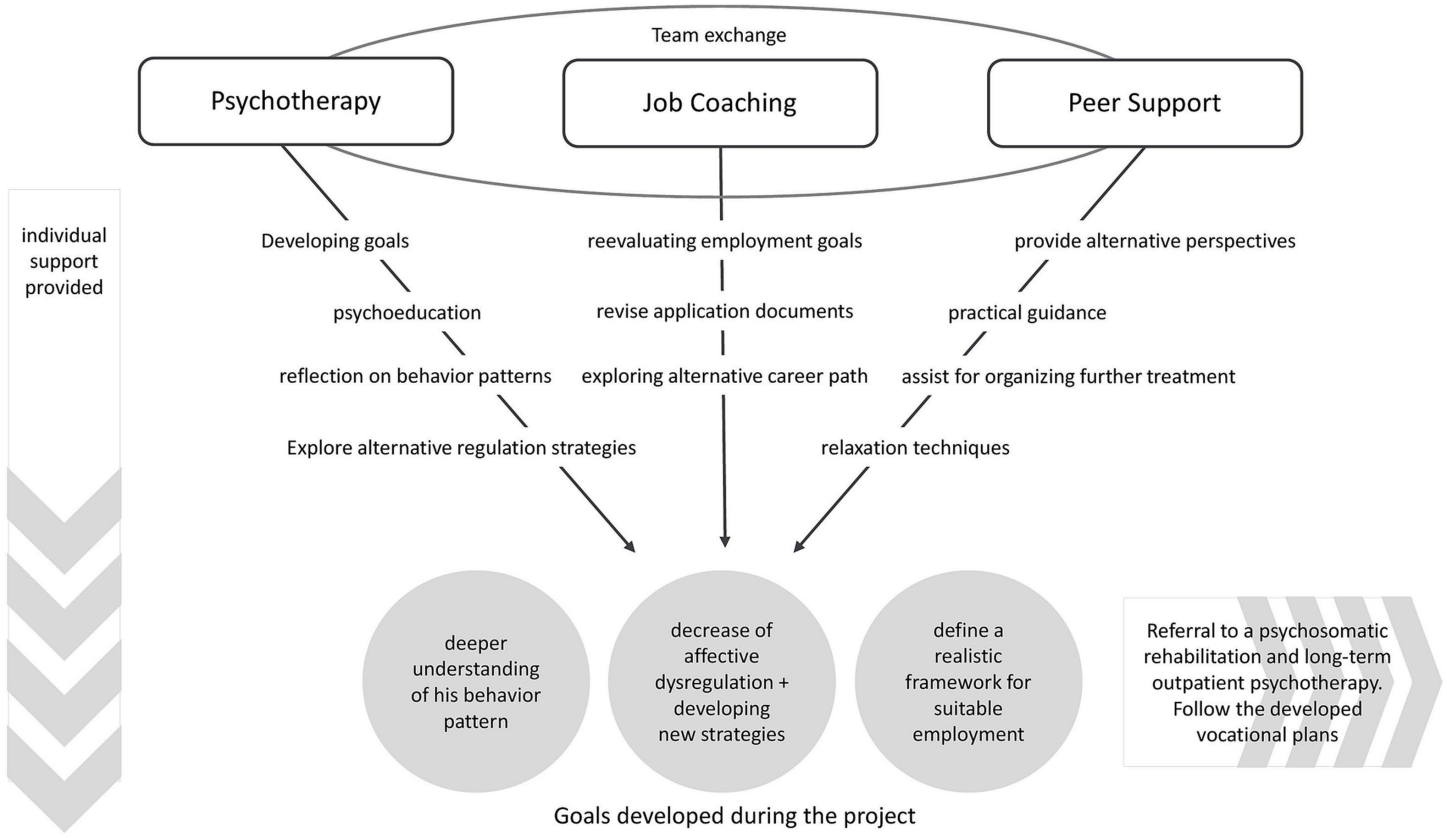
Integrated application of short-term psychotherapy, IPS-based job coaching, and peer support in Participant A.

Participant A initially chose the component of short-term psychotherapy. Her motivation was to “get her things done” and maintain this ability long term, aiming for a meaningful, self-directed life. In session two, peer support was deemed helpful for managing administrative challenges. Peer support began about 1 month after psychotherapy started. Near the end of psychotherapy, she requested job coaching, which began 4 months after enrollment. Peer support and job coaching were initiated based on challenges discussed in therapy or directly requested. Figure 1 illustrates the interaction of short-term psychotherapy, IPS-based job coaching, and peer support and their contribution to key goals in Participant A.

Psychotherapy included ten sessions over 12 months and one booster session. She attended ten planned sessions and one booster. Peer support included 22 contacts. Job coaching totaled 18 sessions.

All providers met weekly to review progress and address issues. Additional informal meetings were held as needed. One joint meeting with all providers and Participant A reassessed goals and support needs. At the peer navigator’s request, a meeting with the psychotherapist and job coach addressed organizational challenges. Due to barriers with the basic income application, the job coach met with local job center staff, who offered targeted support with the application.

The following outlines collaborative efforts to achieve Participant A’s goals or those identified during sessions.

##### 4.1.3.1 Financial issues

In the first psychotherapy session, a comprehensive assessment revealed several stressors, with finances being a central concern. Limited financial resources prevented her from attending psychiatric advised cognitive training, and existential fears made it hard to focus on other areas. Peer support further aimed to stabilize her finances and helped manage related life transitions.

Psychotherapy then focused on identifying personal strengths and social resources. To bridge the financial gap before income support began, she borrowed money from family and friends. Her network also helped to furnish and paint her new apartment - support she found encouraging. Financial hardship was expected to be temporary due to the pending home sale. A short-term plan was created to leverage her strengths, social ties, and interim funding options.

Peer sessions prioritized accelerating the income support process. The peer navigator accompanied her to appointments and helped gather job center related documents. Some administrative tasks, such as locating paperwork, remained difficult (see Section 4.1.3.2). The aim was to empower her in areas she valued. She saw the home sale and divorce progress as key to stability. Psychotherapy and peer work supported her in communicating these needs to legal and personal contacts, though progress was partly out of her control.

Job coaching began near the end of psychotherapy to support employment as a path to psychological stability. Her perseverance and parenting experience were seen as assets. The job search focused on roles in childcare and after-school settings. She started with a mini-job that was not subject to social insurance contributions, with potential for increased hours. Though initial issues arose with punctuality and confusion regarding work locations, she later expanded her role. By 18 months after enrollment, and based on self-report during a follow-up interview, she had secured sufficient working hours to qualify for social security benefits.

##### 4.1.3.2 Improving organizational skills and decision-making

At the beginning of her psychotherapy sessions, Participant A struggled with decision-making and had difficulty identifying goals or important topics. Her background revealed a strained relationship with her mother and limited emotional expression in the family, likely contributing to a lack of emotional markers that can guide decisions (38).

To address this, a psychoeducational approach was used to emphasize that emotional markers aren’t always necessary in decision making. Also, cognitive-behavioral techniques supported structured evaluation of choices, such as weighing pros and cons and using personal values. Mindfulness-based exercises enhanced awareness and tolerance for uncertainty, while scenario-based exercises provided a safe space to practice and build confidence.

Both the peer navigator and the job coach noticed that she often failed to complete tasks like locating documents. These challenges became apparent when Participant A frequently arrived at sessions unprepared, often citing forgetfulness or feeling overwhelmed by the tasks. Recognizing that this behavior was affecting progress across multiple interventions, a joint discussion between providers led to coordinated strategies focused on breaking tasks into clear, manageable steps and fostering accountability.

The peer navigator applied empowerment and scaffolding techniques, helping her create personalized checklists and celebrating small successes to build self-efficacy (39).

The job coach, meanwhile, adopted a pedagogical approach, focusing on teaching practical skills for organization and time management, such as organizing documents with folders and labels, creating a designated space, and using reminders such as texts or calendar alerts tailored to her routine.

In addition to these individual approaches, the peer navigator and job coach worked collaboratively to reinforce skills and strategies. This combination of empowerment, scaffolding, and teaching created a structured support system that improved her ability to manage tasks and supported her broader goals of independence and functioning. Coordination across providers ensured consistent and motivating guidance.

##### 4.1.3.3 Emotional reflection and confrontation

Emotional reflection was addressed across all three intervention components. In psychotherapy, sessions explored the pros and cons of Participant A’s emotional detachment. While detachment was seen as protective in reducing stress or promoting objectivity, exceptions showed that unresolved issues and external pressure still caused emotional strain. This exploration helped her differentiate adaptive and maladaptive emotions.

Peer support built on this by addressing her tendency to avoid emotions and ruminate instead. The peer navigator shared his own experiences to model emotional growth and resilience, offering a safe, relatable space for reflection. This encouraged her to view emotional discomfort as a path to self-awareness rather than something to avoid.

The job coach added a practical angle, linking emotional regulation to job retention. Volatility and distractibility were discussed as possible signs of unprocessed emotions. To increase stability at work, the coach helped her recognize stressors, build daily routines, connect with resources in the community, and prepare for emotionally difficult situations, including how to seek support from supervisors.

Together, the interventions created an integrated approach: psychotherapy offered introspective grounding, peer support added lived experience, and job coaching translated emotional regulation into workplace skills.

### 4.2 Participant B

#### 4.2.1 Participant demographics for Participant B

Participant B is man who in his late 20 s who at the time of enrollment in the 3for1 intervention program lived alone and was in no relationship.

He holds a vocational qualification and has several years of professional experience, along with various additional certifications. In his previous job, he reported experiencing high workloads and a growing sense of overwhelm, which ultimately led to psychological distress and prolonged sick leave, culminating in the termination of his employment. Following this, he was receiving unemployment benefits for a couple of months while being employed in a mini-job arrangement (marginal employment without social security benefits).

Participant B reported having made considerable efforts to secure new employment. However, he expressed concerns about finding a position that would align with both his qualifications and his mental well-being. He was receiving outpatient psychiatric care and was undergoing treatment with antidepressant medication which he perceived as only partially helpful. Despite persistent efforts, he struggled to access appropriate treatment and support prior to entering the 3for1 intervention program.

#### 4.2.2 Diagnostic findings

During the initial assessment, Participant B reported experiencing a persistent sense of inner emptiness and pronounced lack of motivation. Also, he reported intrusive, ruminative thoughts, often accompanied by intense feelings of shame and guilt. He described structuring his daily and nightly routines as cognitively and emotionally demanding, which, among other things, often leads to him missing appointments.

Episodes of binge eating occurred several times per week, followed by self-induced vomiting. These episodes served as a maladaptive strategy for affect regulation, providing temporary relief from emotional emptiness and internal tension. Outside of these episodes, he reported placing a high value on healthy nutrition and frequently engaged in excessive physical exercise. He denied any substance use and reported only occasional alcohol consumption.

#### 4.2.3 Interventions

Participant B’s primary motivation for engaging in the 3for1 intervention program was to reduce his depressive symptoms, improve his ability to structure daily routines, and secure sustainable employment that aligned with both his professional qualifications and emotional well-being. Based on his prior work experience and educational background, he was considered a suitable candidate for reintegration into the primary labor market.

The close collaboration between the intervention components, short-term psychotherapy, job coaching, and peer support was considered a key element of the support process.

Participant B decided to start with the short-term psychotherapy. He attended the maximum of 10 sessions, as well as 3 additional booster sessions.

Four weeks after starting psychotherapy, Participant B requested the assistance of a job coach, utilizing the maximum allowance of 25 sessions.

After completing short-term psychotherapy Participant B also decided to take part in peer support. These sessions were held weekly in low-threshold settings, such as outdoor walks or café meetings. He remained actively engaged in peer support until the end of his intervention period, participating in a total of 13 sessions.

**Figure 2:**
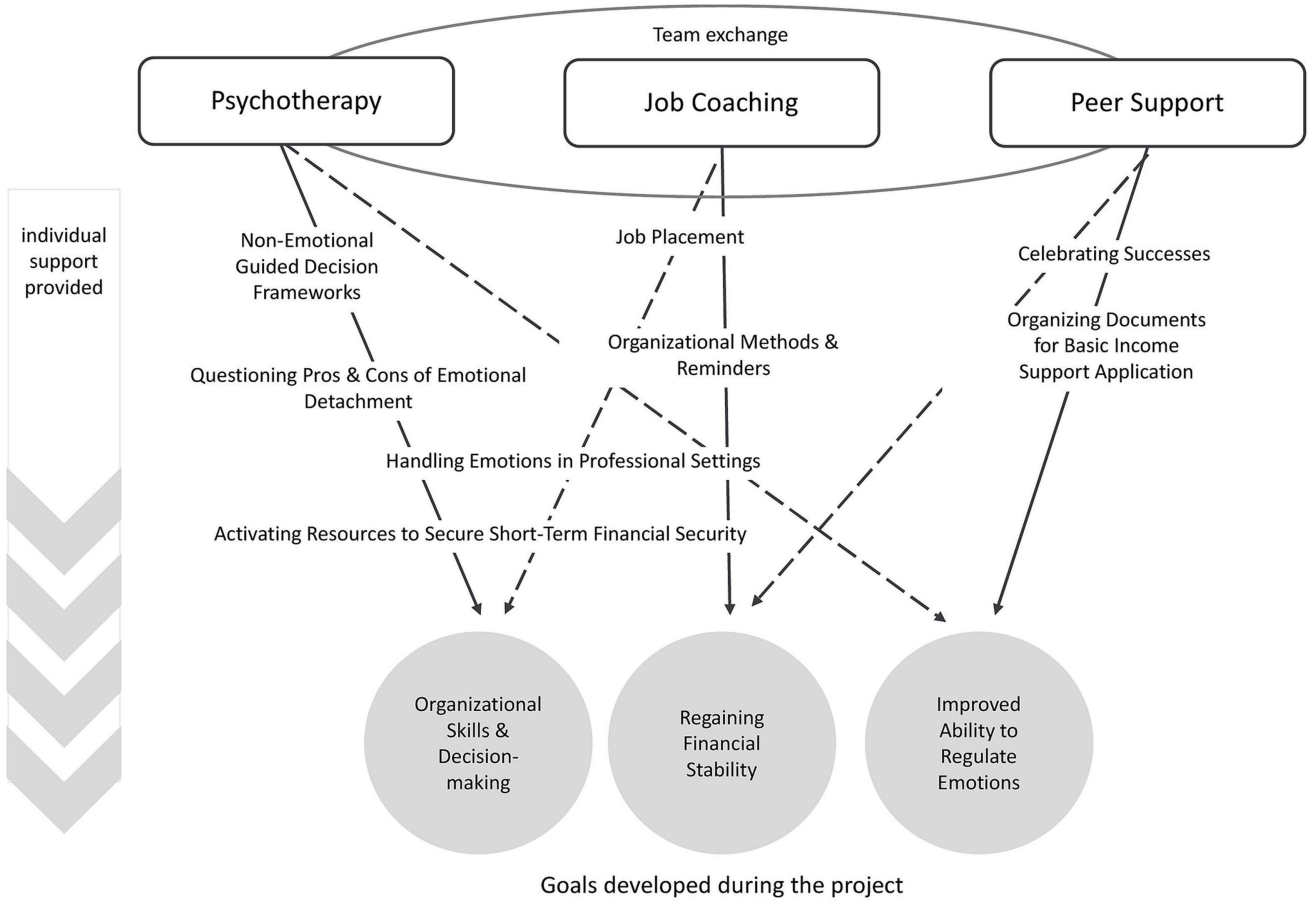
Integrated application of short-term psychotherapy, IPS-based job coaching, and peer support in Participant B.

Although he faced challenges with punctuality, he demonstrated a strong commitment to the program by consistently adhering to the scheduled sessions across all intervention modalities and early difficulties improved over time. The close collaboration between the intervention components was considered a key element of the support process to identify the following key topics and the specific foci of each specific intervention. Figure 2 illustrates the interaction of short-term psychotherapy, IPS-based job coaching, and peer support and their contribution to key goals in Participant B.

##### 4.2.3.1 Self-representation and the struggle for coherence

In the psychotherapy sessions an incongruence between Participant B’s high aspirations for job status and his pronounced anxiety about failing to meet occupational expectations first became apparent. The psychotherapist helped to understand underling patterns as a basis for behavior change. Important biographical factors were his father’s strong emphasis on academic success and aggressive reaction to perceived underperformance. Ongoing family conflicts as well as frequent relocations in childhood forced him to continuously adapt to new social environments. Thus, he developed a highly adaptive self-presentation style to facilitate social integration and acceptance. However, Participant B recognized that this adaptation came at the cost of expressing his own needs and boundaries and asserting himself.

Working with the job coach, the described pattern also became evident. Participant B actively sought to present himself as cooperative and highly motivated. However, vocational tasks, such as revising his already decent CV, elicited significant psychological distress. Due to the integrative approach of the intervention, these issues were explored within short-term psychotherapy, where he was encouraged to discuss his anxieties with the job coach. He recognized that openly addressing his fears and emotions significantly alleviated his distress. Positive feedback from his mini-job employer and initial success in job applications further boosted self-esteem and self-efficacy.

Later in the process the peer support sessions provided a valuable opportunity for him to engage in open dialogue on equal footing. The exchange allowed him to critically examine his core values, explore alternative perspectives, and to cultivate cognitive and emotional flexibility.

In sessions of all three components, Participant B reflected on the significance of social status, contemporary gender norms, and the intricacies of work-life balance. Step by step his rigid perfectionism became less dominant. Over the course of time, his self-concept became more coherent and allowed realistic ideas about future vocational options.

##### 4.2.3.2 Affective regulation and compensatory behaviors

An important factor of Participant B’s psychological distress was his persistent ruminative thinking, which he experienced as highly burdensome. A central focus of the psychotherapy sessions was the collaborative exploration of this vicious cycle. Negative emotions associated with demanding tasks led to procrastination, which in turn triggered feelings of shame and frustration. To alleviate these feelings, Participant B resorted in binge eating, followed by purging or excessive exercise. A key aspect of the therapeutic approach included psychoeducation on the functional role of emotions and the development of alternative emotion regulation strategies. Relaxation techniques and attention focusing techniques were introduced to help him manage physiological arousal and cognitive distress.

The peer support provided both validation and practical guidance concerning the topic. The peer navigator, drawing from her own experience with burnout and sleep disturbances, shared adaptive coping strategies, such as evening walks and structured bedtime rituals. Furthermore, peer support facilitated a shift from avoidance to behavioral activation and therefore self-efficacy, i.e., by helping to organize a move.

##### 4.2.3.3 Reevaluating employment goals in the context of mental health

With the job coach, Participant B was able to establish more realistic expectations for suitable employment. This was facilitated by understanding cognitive-emotional patterns with the help of the psychologist and reflection on his values and expectations with the peer navigator. In the past, he had set high standards regarding job status and salary, frequently terminating positions within a few months when these expectations were unmet or when job demands exceeded his perceived coping capacity. Exploring alternative career paths with the job coach led him recognize that, he still felt fundamentally aligned with his trained profession. He could envision re-entering this field, given that adequate conditions were met, such as part-time employment, limited travel requirements, and a well-structured onboarding process.

However, despite significant progress throughout the 3for1 intervention program and partly remission of the symptoms, the persisting eating disorder symptoms remained a major barrier to transition directly into sustained employment. Through the interventions, a readiness for treatment was established. A referral to an inpatient treatment was recommended, followed by long-term outpatient psychotherapy. Close collaboration with the peer navigator proved invaluable in this phase, as she assisted with the application process for inpatient treatment and the search for a suitable psychotherapist. The subsequent steps were coordinated with the job center and his treating psychiatrist.

After the intervention phase, Participant B reported that he perceived the inpatient treatment as very beneficial and was able to apply for various positions considering the prerequisites, which he had formulated during the project.

## 5 Discussion section that shares practical implications, lessons learned for future applications

The aim of this community case study was to illustrate the implementation and cooperation of the intervention components using the example of two participants and to describe their progress throughout the intervention. The two presented case studies offer an extract of the stressors and strains experienced by individuals who are dependent on financial support from the state. Throughout the intervention period, the complexity and multifaceted nature of the burdens and challenges faced by participants were evident. However, the range of daily hassles often made it difficult to define clear goals together with participants. In some cases, developing a personal goal took up a large part of the project time. The programs multi-level approach was therefore a crucial prerequisite for providing support tailored to these individual needs. The examples illustrate how essential the close cooperation within the program was to develop and maintain a shared focus across all components.

Both participants entered the 3for1 intervention program with different capabilities and difficulties, but in both cases, establishing a trusting working alliance was essential across all interventional components. Only after some times were the participants able to open up and become ready for joint exploration with each intervention provider.

In addition, each intervention component revealed individual challenges that did not necessarily overlap across all areas. The following key lessons emerged, listed separately for each component.

### 5.1 Lessons learned from short-term psychotherapy

During short-term psychotherapy, both participants presented distinct yet overlapping challenges that impacted the therapeutic process.

In the case of Participant A, biographical factors were linked to difficulties in decision-making, emotional awareness and interaction patterns. These factors hindered progress and complicated a focused therapeutic approach. A resource-oriented strategy was used to build self-efficacy by activating social support and strengthening emotional and decision-making capacities. This aligns with findings that focusing too strongly on deficits can feel threatening and disempowering for clients with chronic relational issues (40). Research (41) highlights that such clients’ strengths are often overlooked within therapy and counselling, thereby reinforcing experiences of helplessness.

Participant B attended regular psychological sessions, allowing for an early completion of the short-term therapy. However, emerging topics could no longer be addressed within the initial timeframe. A key issue was his ambivalence towards his binge-eating behavior. Therefore, elements of the Transtheoretical Model of Change were applied. It sees ambivalence as a normal part of change (42), and emphasizes therapeutic dialogue as a means to enhance motivation and resolve ambivalence (43).

The presented cases highlight the importance of flexibility in the structure and duration of short-term psychological support within this setting. In both cases, the standard format of 10 to 13 sessions was not always sufficient to fully address entrenched patterns or ambivalent motivation. Rather than extending psychotherapy per se, the intervention often functioned as an initial step to foster problem awareness, stabilize participants, and enhance motivation for change in order to facilitate engagement with appropriate follow-up treatment or support. This is consistent with a stepped-care perspective, in which short-term interventions serve as a bridge to more intensive or specialized services where needed.

Research suggests those with mild to severe mental health issues often benefit from such tailored and staged approaches (44).

### 5.2 Lessons learned from peer support

Peer support proved especially valuable to help Participant A managing complex stressors, most notably financial insecurity and organizational daily challenges. Through the supportive relationship with a peer navigator, Participant A was able to reflect on her situation, access concrete guidance, and receive low-threshold assistance rooted in shared lived experience. The peer support relationship offered not only practical help but also emotional validation, which appeared to increase her sense of agency in navigating difficult everyday situations.

These findings align with the previous mentioned research (9, 12), which suggests that individuals experiencing unemployment facing mental health challenges benefit most from interdisciplinary care. In Participant A’s case, it was also clear that the in- and outpatient treatment she had received before entering the 3for1 intervention program was unable to meet all of her problem areas. At the same time, these prior treatment experiences may have contributed to the development of problem awareness, readiness for change, and the ability to engage with and benefit from the support offered within the 3for1 intervention program.

In the case of Participant B, it became apparent that emotionally engaging in peer support required a stable, trusting relationship, and above all, time. The more personal nature of peer exchange compared to official support providers like job center staff often means that participants began to open up within the 3for1 intervention program. As a result, time constraints at the end of the program sometimes limited the full potential of the intervention. This insight highlights the importance of early exposure to peer support within such programs, allowing participants to make informed decisions about if and when to engage with this component. Both peer navigators as well as participants benefit from clarity in peer support roles (45) as well as adequate time to build trust (46). This is in line with findings from recent systematic reviews and meta-analyses on peer support interventions which consistently highlight relational processes, as well as mechanisms such as hope and empowerment, as central to the effectiveness of peer support (37, 47). Therefore, an early introduction to peer support can be vital to initiate a process of change.

### 5.3 Lessons learned from IPS-based job coaching

The job coaching approach emphasized the strengths and aspirations of the participants, following the principles of the Individual Placement and Support (IPS) model.

For Participant A, this meant securing a mini-job in the childcare sector. Although the position was not subject to social security contributions, the arrangement was appropriate given her personal circumstances and the program’s structural limitations. This case supports the findings that flexible employment models are effective for individuals experiencing psychological distress (48). In line with the IPS principle of “first place, then train” (4), the needs and goals analysis revealed that further training was unnecessary. Participant A already possessed the skills required for the role. This underscores the efficacy of a resource-oriented coaching approach that leverages existing competencies rather than focusing on deficits.

Participant B entered the program with strong qualifications and initially demonstrated high levels of engagement. However, over time it became clear that there were psychological and emotional barriers impeding rapid job placement. Through a trusting coaching relationship, he was gradually able to reflect more realistically on the type of job that would suit his needs and capabilities. This self-reflection led to the acknowledgment that a temporary withdrawal from the job market, via inpatient treatment, was a necessary step before re-entering the first labor market. Participant B’s trajectory highlights the importance of respecting this readiness and of adjusting the job search so that a stable long-term employment becomes more likely.

## 6 Strengths, limitations, and conclusion

The presented community case study illustrates the impact of support structures for individuals experiencing unemployment and psychological distress by helping them navigate challenges and reenter the labor market. Collaborative discussions among providers proved particularly beneficial. More specifically, regular interdisciplinary meetings and structured team exchange, as well as case-specific exchanges, were used to coordinate the different intervention components, identify emerging challenges, and adjust support strategies accordingly. For example, difficulties in emotional regulation, which became apparent in one component, could be addressed jointly and translated into concrete strategies across psychotherapy, job coaching, and peer support.

These discussions and team exchanges facilitated shared goal-setting, ensured consistency in the guidance provided to participants, and allowed for timely and coordinated responses to individual barriers. The interaction between components, as illustrated in Figures 1, 2, reflects how these collaborative processes contributed to individualized and adaptive support.

However, limitations remain. The upper limit of 10 + 3 psychotherapy session was often found insufficient for participants with complex health issues or persistent interpersonal patterns. Joint meetings among psychologists, job coaches, and peer navigators revealed that some difficulties, such as those seen in Participant A’s case, surfaced more clearly in specific components like peer support or job coaching. Close interdisciplinary communication remains essential for identifying and addressing such barriers collaboratively. Although regular meetings were built into the program, coordination could have been improved through more intensive shared goal-setting and stronger collaboration. In addition, collaboration with the broader support system was at times challenging and revealed room for improvement, particularly regarding responsibilities and coordination across institutions. This was especially evident for unemployed individuals with mental health conditions, whose needs often span multiple systems, including employment services, health care, and social welfare. While the 3for1 intervention program aimed to provide low-threshold and coordinated support, challenges became especially apparent when participants required services beyond the project. In particular, navigating the interfaces between job centers, health insurance providers, pension insurance, and mental health services proved difficult. Responsibilities were often unclear, services were not easily accessible, and bureaucratic procedures posed substantial barriers, for example in accessing outpatient psychotherapy. As a result, both participants and intervention providers frequently required active effort to navigate the system, including attempts to contact services despite the absence of clearly defined contact persons or responsibilities. These challenges highlight structural gaps in the support system, where individuals with complex needs, as well as the professionals supporting them, may struggle to coordinate care across institutions with differing mandates.

While no *one size-fits all solution* should be derived from the present study, the findings underline the need for more integrated care pathways, clearer allocation of responsibilities, and more accessible, low-threshold points of contact tailored to the needs of unemployed individuals with mental health conditions and the professionals involved in their care.

At the same time, the strengths of the 3for1 intervention program lie in its provision of low-threshold, needs-based support without waiting times. As outlined by the two case studies, this intervention program may help across various problem areas, including accompanying individuals to appointments, particularly for those encountering challenges related to paperwork or language barriers.

However, the intervention program also encountered structural limitations which could not be circumvented within the scope of the project-inherent implementation. It lacked the capacity to provide high-frequency, intensive, or pharmacologically supported interventions for individuals with severe and chronic mental health conditions - needs that are common in this target population. Broader system gaps, like long waiting times for inpatient care or limited outpatient access, constrained its overall effect. The 3for1 intervention program was originally planned as a means of prevention, as outlined in the study protocol (1). Some participants were already too unwell for preventive measures to be effective. Reentry into the primary labor market, as in the case of Participant B, first required more intensive curative treatment.

## Data Availability

The original contributions presented in the study are included in the article/supplementary material, further inquiries can be directed to the corresponding author.
